# Supplementary material for: Contribution of temporal data to predictive performance in 30-day readmission of morbidly obese patients
Source: PeerJ. 2017 Apr 25;5:e3230. doi: 10.7717/peerj.3230 (PMC5407280; doi:10.7717/peerj.3230)
Supplement: Supplemental Information 1 [file peerj-05-3230-s001.pdf]

Lasso Logistic Regression

Dataset	Number of historical records	AUC	95% CI	Brier	95% CI	Sensitivity	95% CI	PPV	95% CI	Specificity	95% CI	NPV	95% CI	NumSelectedMin	95% CI
D1	1	0.639	(0.637, 0.642)	0.212	(0.211, 0.213)	0.57	(0.564, 0.576)	0.448	(0.445, 0.452)	0.632	(0.626, 0.638)	0.738	(0.735, 0.74)	187.17	(179.257, 195.083)
D2	1	0.65	(0.648, 0.653)	0.211	(0.21, 0.212)	0.594	(0.588, 0.601)	0.456	(0.453, 0.46)	0.623	(0.616, 0.63)	0.743	(0.74, 0.746)	169.72	(166.629, 172.811)
	2	0.673	(0.67, 0.675)	0.207	(0.206, 0.207)	0.629	(0.623, 0.634)	0.475	(0.472, 0.478)	0.63	(0.624, 0.636)	0.762	(0.759, 0.764)	158.13	(153.233, 163.027)
D3	1	0.64	(0.637, 0.643)	0.219	(0.218, 0.22)	0.589	(0.582, 0.596)	0.473	(0.468, 0.477)	0.613	(0.605, 0.62)	0.718	(0.714, 0.721)	108.7	(105.242, 112.158)
	2	0.661	(0.658, 0.664)	0.215	(0.214, 0.216)	0.625	(0.617, 0.633)	0.49	(0.485, 0.494)	0.617	(0.61, 0.623)	0.737	(0.733, 0.741)	130.04	(123.352, 136.728)
	3	0.672	(0.669, 0.675)	0.213	(0.212, 0.214)	0.621	(0.613, 0.628)	0.506	(0.502, 0.511)	0.643	(0.636, 0.65)	0.743	(0.74, 0.746)	138.95	(133.1, 144.8)
D4	1	0.625	(0.621, 0.628)	0.228	(0.227, 0.229)	0.582	(0.573, 0.59)	0.495	(0.49, 0.5)	0.601	(0.593, 0.61)	0.682	(0.678, 0.687)	88.34	(80.673, 96.007)
	2	0.65	(0.647, 0.654)	0.224	(0.223, 0.225)	0.616	(0.608, 0.625)	0.518	(0.512, 0.523)	0.614	(0.606, 0.623)	0.706	(0.701, 0.71)	82.39	(73.7, 91.08)
	3	0.665	(0.661, 0.668)	0.22	(0.219, 0.221)	0.627	(0.619, 0.635)	0.528	(0.523, 0.533)	0.623	(0.615, 0.631)	0.714	(0.71, 0.718)	115.33	(108.193, 122.467)
	4	0.668	(0.664, 0.671)	0.22	(0.219, 0.221)	0.631	(0.623, 0.639)	0.532	(0.527, 0.537)	0.627	(0.62, 0.634)	0.718	(0.713, 0.722)	69.28	(64.12, 74.44)
D5	1	0.631	(0.627, 0.636)	0.232	(0.232, 0.233)	0.578	(0.568, 0.588)	0.544	(0.538, 0.551)	0.62	(0.609, 0.632)	0.654	(0.648, 0.659)	29.52	(25.264, 33.776)
	2	0.657	(0.652, 0.661)	0.228	(0.227, 0.229)	0.628	(0.618, 0.638)	0.562	(0.556, 0.567)	0.617	(0.608, 0.626)	0.681	(0.675, 0.687)	32.95	(30.472, 35.428)
	3	0.67	(0.665, 0.674)	0.225	(0.224, 0.226)	0.627	(0.617, 0.636)	0.575	(0.569, 0.581)	0.637	(0.628, 0.646)	0.687	(0.682, 0.693)	47.85	(44.78, 50.92)
	4	0.674	(0.669, 0.679)	0.224	(0.223, 0.225)	0.641	(0.632, 0.65)	0.575	(0.569, 0.581)	0.628	(0.618, 0.639)	0.692	(0.686, 0.698)	42.8	(39.861, 45.739)
	5	0.678	(0.673, 0.683)	0.223	(0.222, 0.224)	0.644	(0.636, 0.653)	0.578	(0.572, 0.584)	0.631	(0.622, 0.641)	0.695	(0.69, 0.701)	54.98	(51.864, 58.096)

XGBoost

Dataset	Number of historical records	AUC	95% CI	Brier	95% CI	Sensitivity	95% CI	PPV	95% CI	Specificity	95% CI	NPV	95% CI	NumSelectedMin	95% CI
D1	1	0.645	(0.642, 0.647)	0.21	(0.21, 0.211)	0.581	(0.577, 0.584)	0.441	(0.438, 0.445)	0.615	(0.612, 0.619)	0.737	(0.734, 0.74)	16.63	(16.312, 16.948)
D2	1	0.637	(0.635, 0.639)	0.21	(0.21, 0.211)	0.593	(0.586, 0.6)	0.434	(0.431, 0.437)	0.587	(0.579, 0.596)	0.731	(0.728, 0.733)	17.13	(16.88, 17.38)
	2	0.673	(0.67, 0.675)	0.207	(0.207, 0.208)	0.59	(0.584, 0.596)	0.476	(0.472, 0.48)	0.654	(0.647, 0.66)	0.75	(0.748, 0.753)	20.21	(19.907, 20.513)
D3	1	0.634	(0.631, 0.637)	0.215	(0.215, 0.216)	0.598	(0.588, 0.609)	0.453	(0.449, 0.457)	0.574	(0.563, 0.585)	0.709	(0.705, 0.713)	19.21	(18.934, 19.486)
	2	0.668	(0.665, 0.671)	0.213	(0.212, 0.213)	0.591	(0.583, 0.599)	0.5	(0.494, 0.505)	0.65	(0.64, 0.66)	0.73	(0.727, 0.734)	22.04	(21.677, 22.403)
	3	0.675	(0.672, 0.679)	0.211	(0.21, 0.212)	0.579	(0.572, 0.585)	0.513	(0.508, 0.519)	0.677	(0.67, 0.683)	0.732	(0.729, 0.736)	23.6	(23.287, 23.913)
D4	1	0.623	(0.62, 0.627)	0.221	(0.22, 0.221)	0.507	(0.493, 0.52)	0.498	(0.491, 0.504)	0.652	(0.638, 0.667)	0.664	(0.66, 0.669)	19.31	(18.996, 19.624)
	2	0.665	(0.661, 0.669)	0.218	(0.217, 0.219)	0.588	(0.58, 0.597)	0.533	(0.527, 0.538)	0.652	(0.643, 0.662)	0.703	(0.699, 0.707)	23.11	(22.738, 23.482)
	3	0.68	(0.677, 0.684)	0.216	(0.215, 0.216)	0.608	(0.6, 0.617)	0.55	(0.543, 0.556)	0.664	(0.654, 0.673)	0.717	(0.713, 0.721)	22.76	(22.446, 23.074)
	4	0.686	(0.682, 0.689)	0.215	(0.215, 0.216)	0.619	(0.61, 0.628)	0.547	(0.541, 0.554)	0.655	(0.645, 0.665)	0.72	(0.716, 0.724)	22.21	(21.835, 22.585)
D5	1	0.638	(0.632, 0.643)	0.226	(0.225, 0.227)	0.524	(0.503, 0.545)	0.549	(0.54, 0.557)	0.655	(0.633, 0.676)	0.641	(0.635, 0.647)	18.72	(18.412, 19.028)
	2	0.675	(0.67, 0.68)	0.222	(0.222, 0.223)	0.623	(0.611, 0.636)	0.571	(0.565, 0.576)	0.632	(0.62, 0.644)	0.684	(0.679, 0.69)	22.01	(21.613, 22.407)
	3	0.685	(0.68, 0.691)	0.222	(0.221, 0.223)	0.618	(0.607, 0.629)	0.583	(0.577, 0.589)	0.655	(0.645, 0.664)	0.688	(0.682, 0.694)	23.51	(23.14, 23.88)
	4	0.684	(0.679, 0.689)	0.222	(0.221, 0.223)	0.625	(0.613, 0.637)	0.585	(0.578, 0.591)	0.651	(0.638, 0.663)	0.691	(0.685, 0.697)	23.89	(23.499, 24.281)
	5	0.686	(0.681, 0.691)	0.222	(0.221, 0.223)	0.625	(0.613, 0.637)	0.58	(0.574, 0.587)	0.645	(0.633, 0.656)	0.689	(0.683, 0.696)	23.9	(23.518, 24.282)

Random Forest

Dataset	Number of historical records	AUC	95% CI	Brier	95% CI	Sensitivity	95% CI	PPV	95% CI	Specificity	95% CI	NPV	95% CI	NumSelectedMin	95% CI
D1	1	0.655	(0.653, 0.658)	0.231	(0.23, 0.231)	0.597	(0.592, 0.603)	0.445	(0.442, 0.449)	0.61	(0.604, 0.616)	0.743	(0.741, 0.746)	313.82	(311.728, 315.912)
D2	1	0.648	(0.645, 0.65)	0.232	(0.232, 0.232)	0.584	(0.577, 0.592)	0.447	(0.443, 0.45)	0.614	(0.606, 0.622)	0.736	(0.733, 0.738)	242.59	(241.314, 243.866)
	2	0.678	(0.675, 0.68)	0.23	(0.229, 0.23)	0.604	(0.597, 0.61)	0.475	(0.472, 0.479)	0.645	(0.637, 0.652)	0.754	(0.752, 0.756)	322.69	(320.853, 324.527)
D3	1	0.646	(0.643, 0.649)	0.235	(0.235, 0.235)	0.577	(0.569, 0.585)	0.472	(0.467, 0.476)	0.619	(0.611, 0.628)	0.714	(0.71, 0.718)	232.18	(231.05, 233.31)
	2	0.674	(0.67, 0.677)	0.233	(0.233, 0.233)	0.605	(0.597, 0.612)	0.505	(0.5, 0.51)	0.65	(0.641, 0.658)	0.737	(0.733, 0.74)	324.92	(322.848, 326.992)
	3	0.686	(0.683, 0.69)	0.232	(0.232, 0.232)	0.603	(0.597, 0.61)	0.518	(0.513, 0.524)	0.67	(0.663, 0.676)	0.742	(0.738, 0.745)	317.8	(315.655, 319.945)
D4	1	0.64	(0.637, 0.644)	0.238	(0.238, 0.239)	0.573	(0.564, 0.582)	0.501	(0.495, 0.506)	0.615	(0.605, 0.625)	0.683	(0.679, 0.688)	230.47	(229.301, 231.639)
	2	0.679	(0.676, 0.683)	0.236	(0.236, 0.236)	0.625	(0.616, 0.634)	0.54	(0.535, 0.546)	0.643	(0.634, 0.652)	0.72	(0.715, 0.724)	326.58	(324.607, 328.553)
	3	0.69	(0.686, 0.693)	0.235	(0.235, 0.235)	0.615	(0.609, 0.622)	0.556	(0.549, 0.562)	0.669	(0.661, 0.677)	0.722	(0.718, 0.726)	321.83	(319.897, 323.763)
	4	0.694	(0.691, 0.698)	0.235	(0.235, 0.235)	0.619	(0.612, 0.627)	0.559	(0.553, 0.564)	0.671	(0.664, 0.678)	0.725	(0.721, 0.729)	319.95	(318.237, 321.663)
D5	1	0.647	(0.643, 0.652)	0.241	(0.241, 0.242)	0.595	(0.584, 0.606)	0.544	(0.538, 0.551)	0.61	(0.599, 0.621)	0.66	(0.654, 0.665)	216.94	(215.794, 218.086)
	2	0.681	(0.676, 0.686)	0.239	(0.239, 0.239)	0.625	(0.616, 0.634)	0.581	(0.575, 0.586)	0.647	(0.638, 0.655)	0.689	(0.684, 0.695)	311.26	(309.272, 313.248)
	3	0.69	(0.684, 0.695)	0.239	(0.238, 0.239)	0.619	(0.61, 0.628)	0.596	(0.589, 0.602)	0.67	(0.661, 0.68)	0.693	(0.688, 0.699)	311.79	(309.837, 313.743)
	4	0.692	(0.687, 0.697)	0.238	(0.238, 0.239)	0.635	(0.626, 0.644)	0.592	(0.586, 0.599)	0.658	(0.649, 0.667)	0.698	(0.693, 0.704)	315.35	(313.684, 317.016)
	5	0.696	(0.691, 0.701)	0.238	(0.238, 0.239)	0.641	(0.631, 0.65)	0.592	(0.586, 0.598)	0.655	(0.646, 0.663)	0.701	(0.695, 0.707)	311.86	(310.218, 313.502)
