# Supplementary figures and images for: Contribution of temporal data to predictive performance in 30-day readmission of morbidly obese patients
Source: PeerJ. 2017 Apr 25;5:e3230. doi: 10.7717/peerj.3230 (PMC5407280; doi:10.7717/peerj.3230)

Supplement 2. Performance of Random Forest predictive models.

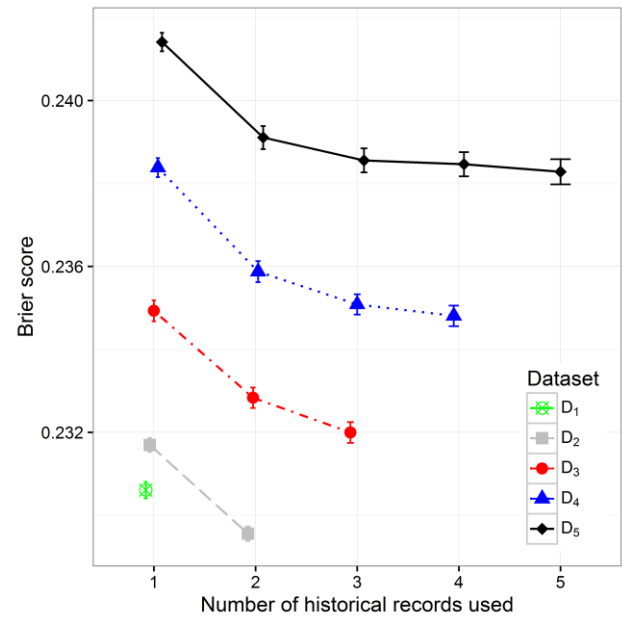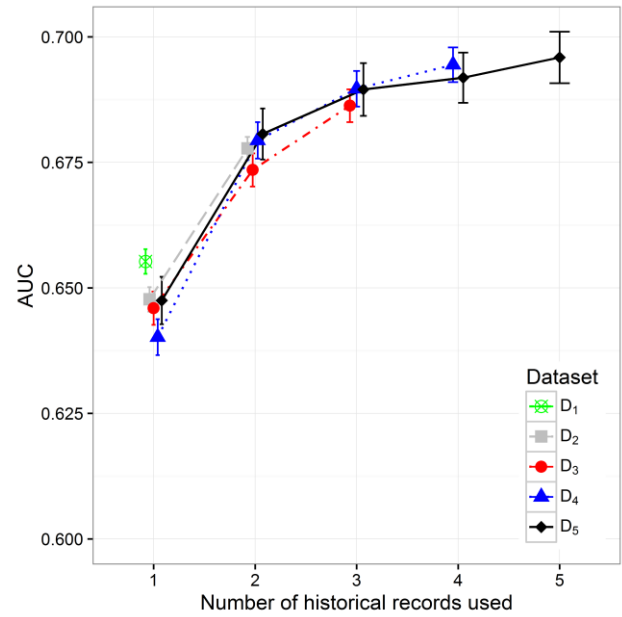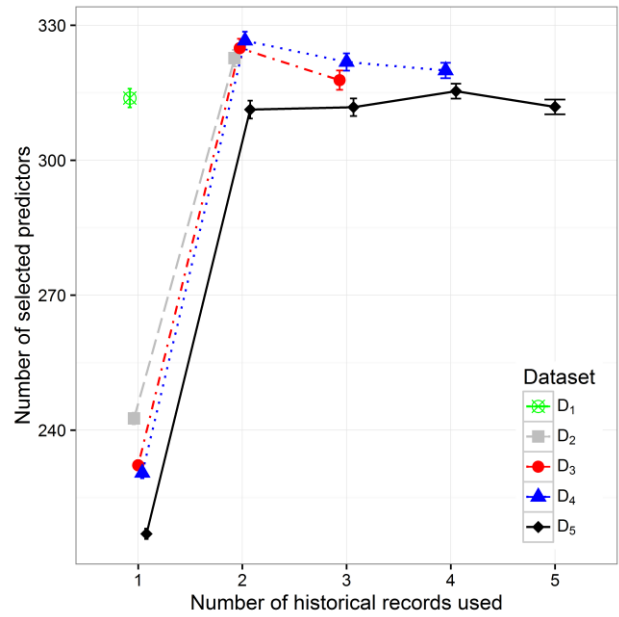

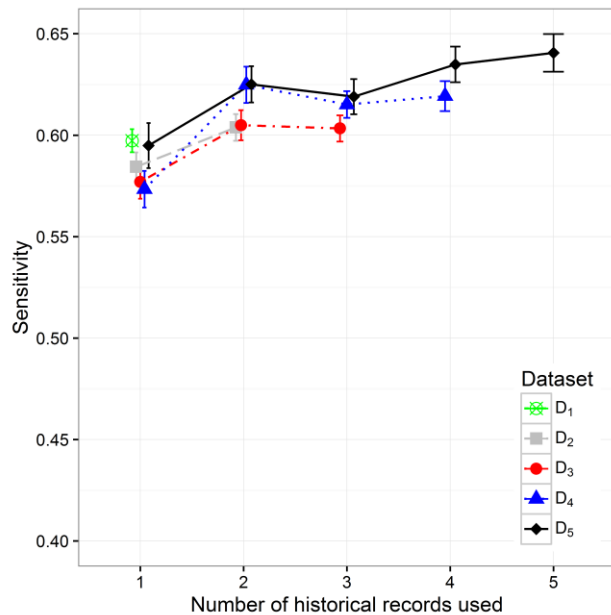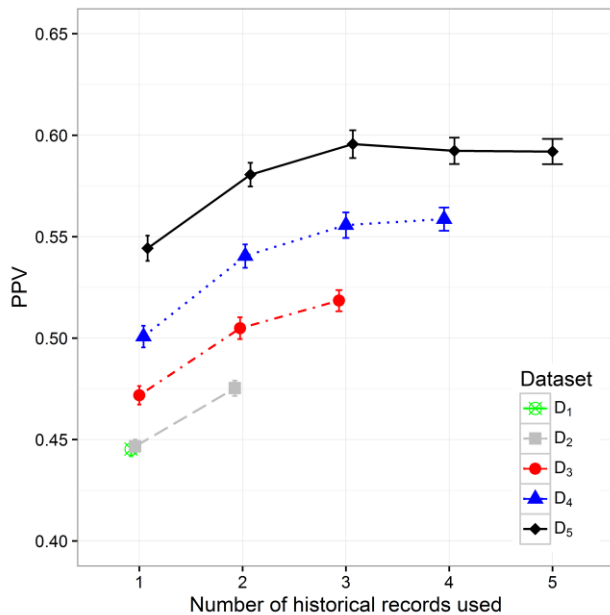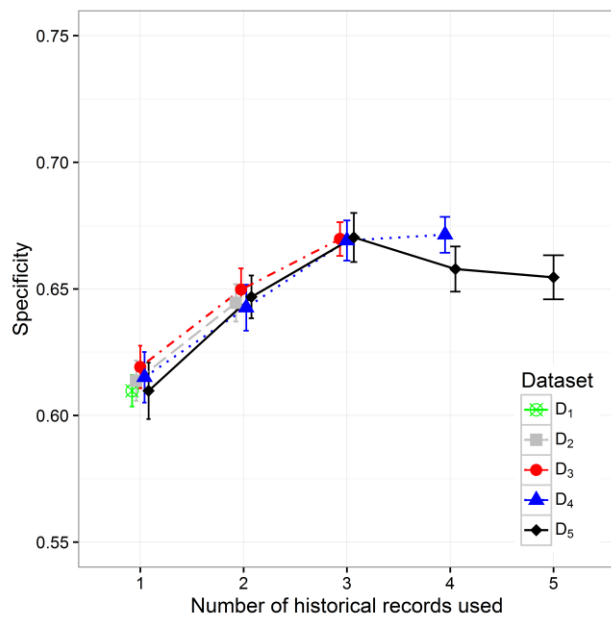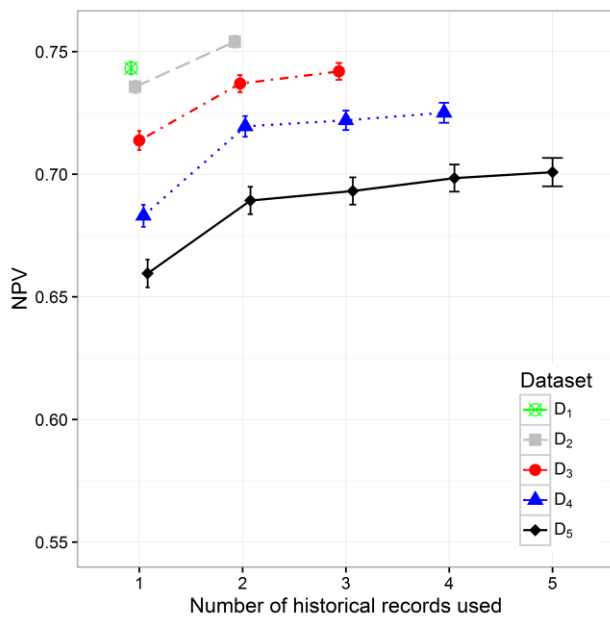

Supplement: Supplemental Information 2 [file peerj-05-3230-s002.pdf]

*Supplement 3. Performance of XGBoost predictive models.*

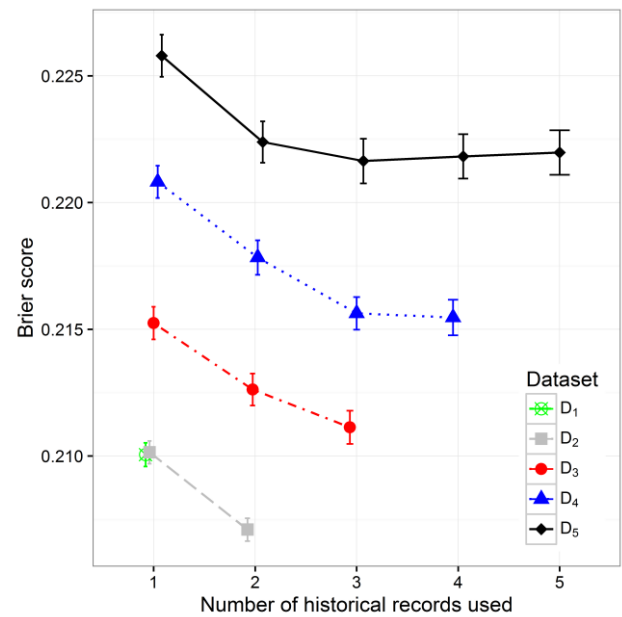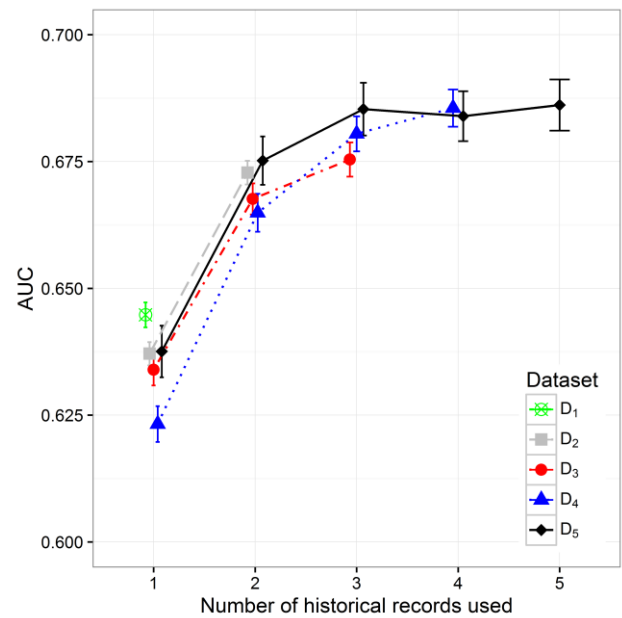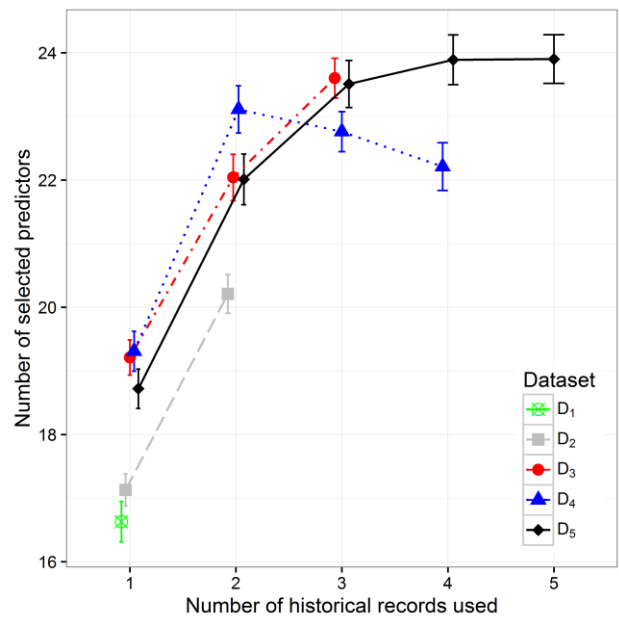

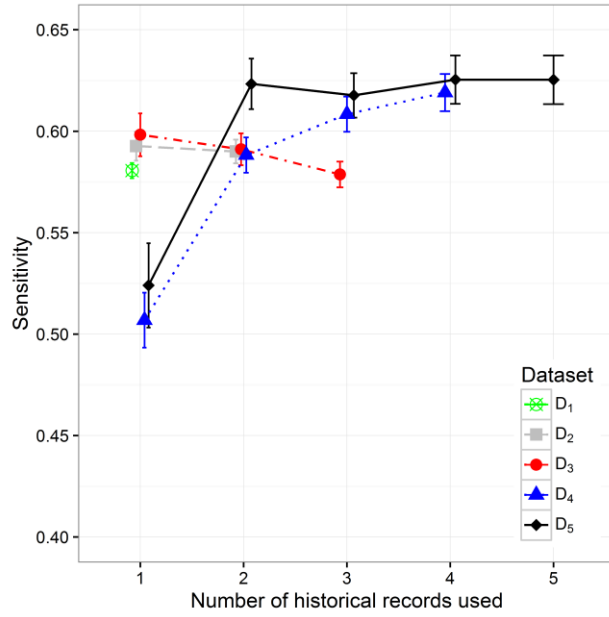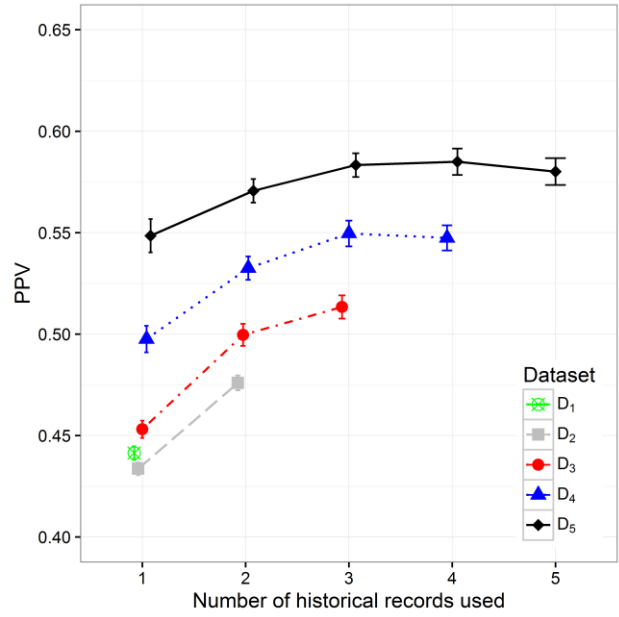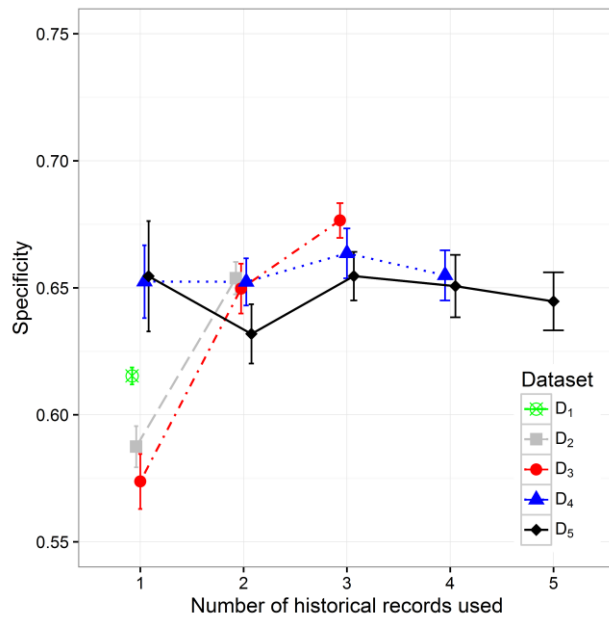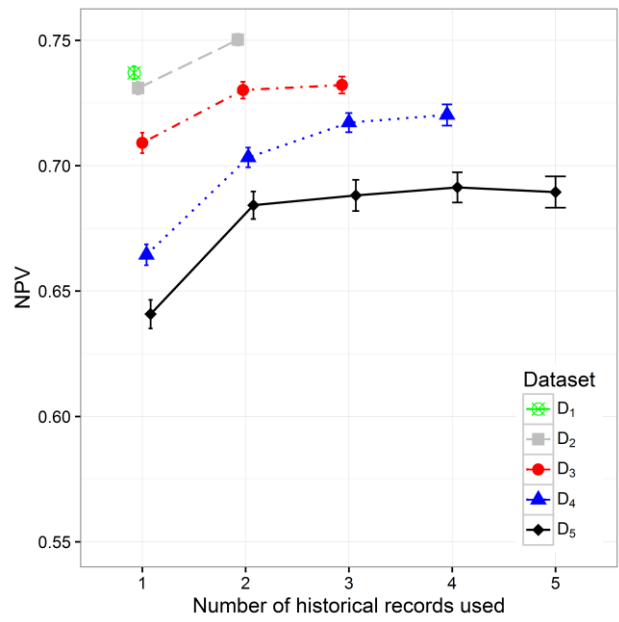

Supplement: Supplemental Information 3 [file peerj-05-3230-s003.pdf]
